# ﻿An additional ✝Archearadinae flat-bug species from Cretaceous Burmese Amber (Hemiptera, Aradidae)

**DOI:** 10.3897/zookeys.1219.137409

**Published:** 2024-11-28

**Authors:** Royce T. Cumming, Julia J. Mlynarek

**Affiliations:** 1 Montreal Insectarium, 4581 rue Sherbrooke est, Montréal, H1X 2B2, Québec, Canada Montreal Insectarium Montreal Canada; 2 Richard Gilder Graduate School, American Museum of Natural History, New York, NY 10024, USA Richard Gilder Graduate School, American Museum of Natural History New York United States of America; 3 Biology, Graduate Center, City University of New York, New York, USA City University of New York New York United States of America

**Keywords:** Burma, Cenomanian, extinct, Heteroptera, Myanmar, new species, Pentatomomorpha

## Abstract

Currently 19 species of Aradidae (flat bugs) are known from the Cretaceous deposits of Burma (Burmese/Kachin amber). In reviewing unidentified aradid species from this deposit, an unnamed species was located. This aradid includes a unique combination of features from several Cretaceous aradid genera coupled with apomorphic antennae morphology allows easy differentiation from other aradids. Therefore, a new genus and species is herein described as *Sauronaradusmeganae***gen. et. sp. nov.** to accommodate these unique features.

## ﻿Introduction

The Aradidae is a cosmopolitan family of true bugs with more than 2000 described species in 8 subfamilies and 230 genera (Schuh and Slater 1995). The Aradidae are considered most diverse in Australia but can be found in all biogeographic realms except Antarctica (Schuh and Slater 1995). They are easily recognizable due to their dorsoventrally flattened bodies and rough bark-like texture. Aradid species can be macropterous or apterous. Even though their ecology is not well documented, they are believed to be mycophagous and individuals can generally be found living under bark of dead trees, on fallen branches, or in the leaf litter (Swanson 2020) while some species are associated with clear cutting and forest fires (Deyrup and Mosley 2004; Johansson et al. 2010).

Species of Aradidae are somewhat prevalent in the fossil record. Thirty-nine extinct species have been described from various fossil deposits from the Cretaceous to the present. Deposits include middle-late Eocene Baltic amber (Heiss 2000; Heiss 2002a, b, c), Lower Eocene French amber (Marchal et al. 2011), early to mid-Miocene Dominican amber (Froeschner 1992), early to mid-Miocene Mexican amber (Heiss 2016), Miocene Shangwang formation in China (Zhang et al. 1994), Early Miocene Foulden Maar formation in New Zealand (Kaulfuss et al. 2011) and from mid-Cretaceous Burmese/Kachin amber (Heiss and Grimaldi 2001, 2002; Grimaldi et al. 2002).

Kachin amber (found in the Hukawng Basin of Kachin State in northern Myanmar) dates from the mid-Cretaceous around 100 million years ago (Shi et al. 2012). Kachin amber is considered especially important because of the diverse fauna it has preserved from a crucial period in the Cretaceous (Ross 2024). This amber deposit has also been prolific for aradids in particular. Currently there are 19 species from 12 genera of Aradidae described from Kachin amber (Heiss and Chen 2023b; Heiss 2023). In 2001, Aradidae from Kachin amber began to be described and Heiss and Grimaldi (2001) described two species of *Archearadus* Heiss & Grimaldi, 2001. Grimaldi and Engel (2008) described the new genus, *Cretopiesma*. In 2012, one species of *Myanmezira* Heiss & Poinar, 2012 and one species of *Kachinocoris* Heiss, 2012 were described (Heiss and Poinar 2012; Heiss 2012). Heiss (2016, 2019b, 2019c) added a further three more species, one each of *Aradoleptus* Heiss, 2016, *Archaeneurus* Heiss, 2019a and *Archecalisius* Heiss, 2019a. In 2020, Azar et al. (2020) revised *Cretopiesma* Grimaldi & Engel, 2008 from Kachin amber and described three new species. In 2022, Heiss (2022) described the new genus *Pachytylaradus*. Most recently in 2023, Heiss (2023) described a new species of *Cretozemira* Heiss, 2023 and Heiss and Chen (2023a, 2023b) described two species belonging to the new genus *Archemezira* Heiss & Chen, 2023.

We describe an additional new genus and species in this remarkable family of true bugs belonging to the extinct subfamily ✝Archearadinae from Kachin amber.

## ﻿Material and methods

The amber containing the holotype specimen is from the well-known Hukawng Valley in northern Myanmar, a prolific site of amber excavation (Grimaldi et al. 2002). The age of this amber deposit is estimated to be ~98.79 ± 0.62 million years old, within the Cenomanian stage of the Cretaceous (Shi et al. 2012). The specimen described and illustrated herein was morphologically reviewed using a 2x-225x trinocular boom stand stereo microscope (#ZM-4TW3-FOR-20MBI3) and photographs were taken with an attached high-speed 20MP camera (#MU2003-BI-CK) (AmScope, Irvine, USA). Illumination was from a 6-Watt LED dual gooseneck illuminator lit by a #85-265VAC/50-60Hz lighting unit (AmScope, Irvine, USA). Measurements were taken using an AmLite digital camera software for Mac OS X 10.8 64-bit which was calibrated with a 0.01 mm microscope stage calibration slide (#MR095) (AmScope, Irvine, USA). Adobe Photoshop Elements 13 (Adobe Inc., San Jose, USA) was used as post-processing software.

Aradidae head morphology terminology follows Rakitov (2022).

## ﻿Aradidae currently described from Burmese Amber

### ﻿†Archearadinae Heiss & Grimaldi, 2002

*Archearadusburmensis* Heiss & Grimaldi, 2001

*Archearaduselongatus* Heiss, 2016

*Archemeziranuoxichenae* Heiss & Chen, 2023^*^

*Archemeziranuoyichenae* Heiss & Chen, 2023^*^

*Cretopiesmaanticum* (Heiss & Poinar, 2012)^**^

*Cretopiesmaengelgrimaldii* Azar, Heiss & Huang, 2020

*Cretopiesmainexpectatum* Azar, Heiss & Huang, 2020

*Cretopiesmalini* Azar, Heiss & Huang, 2020

*Cretopiesmasuukyiae* Grimaldi & Engel, 2008^***^

*Cretozemiraelongata* Heiss, 2023

*Sauronaradusmeganae* gen. et. sp. nov.^*^

### ﻿Mezirinae Oshanin 1908

*Myanmeziralongicornis* Heiss & Poinar, 2012

### ﻿Calisiinae Stål 1873

*Calisiomorphayuripopovi* Heiss, 2016^*^

*Calisiomorphaherczeki* Heiss, 2023

*Archecalisiuslongiventris* Heiss, 2019

### ﻿Unspecified subfamily

*Aradoleptusbirmanus* Heiss, 2016

*Ellenbergeriaoviventris* Heiss, 2016

*Kachinocorisbrevipennis* Heiss, 2012

*Pachytylaraduscretaceous* Heiss, 2022

*Archeaneurusneli* Heiss, 2019

## ﻿Systematic palaeontology

**Order Hemiptera Linnaeus, 1758**

**Suborder Heteroptera Latreille, 1810**

**Infraorder Pentatomomorpha Leston, Pendergrast & Southwood, 1954**

**Family Aradidae Brullé, 1836**

**Subfamily** ✝**Archearadinae Heiss & Grimaldi, 2002 (tentatively placed)**

### ﻿Genus Sauronaradus gen. nov.

Taxon classificationAnimaliaHemipteraAradidae

C74ED450-7625-5712-A777-98781C11E929

https://zoobank.org/653B62FB-8028-4D63-9ABA-0B17FB4F2037

#### Type species.

*Sauronaradusmeganae* gen. et sp. nov., herein designated.

#### Etymology.

Generic name derived from the epic fantasy novels “The Lord of the Rings” by J. R. R. Tolkien (1892–1973). Both authors independently, upon seeing the spines and armor-like habitus of this species, thought of the armored cinematic depiction of the villainous protagonist Sauron during the “War of the Last Alliance” during the “late Second Age”. The name Sauron ([ˈsaʊron] or [ˈθaʊron], is from the language “Quenya” [one of the languages spoken by the High Elves of Middle-earth]), and he is the eponymous “Lord of the Rings”. The eponym is coupled with á*rados* (Greek: ἄρᾰδος), which is Latinized as “*aradus*”, referencing the relationship to Aradidae. Gender is masculine following -*aradus*.

#### Diagnosis.

**Figure 1.**
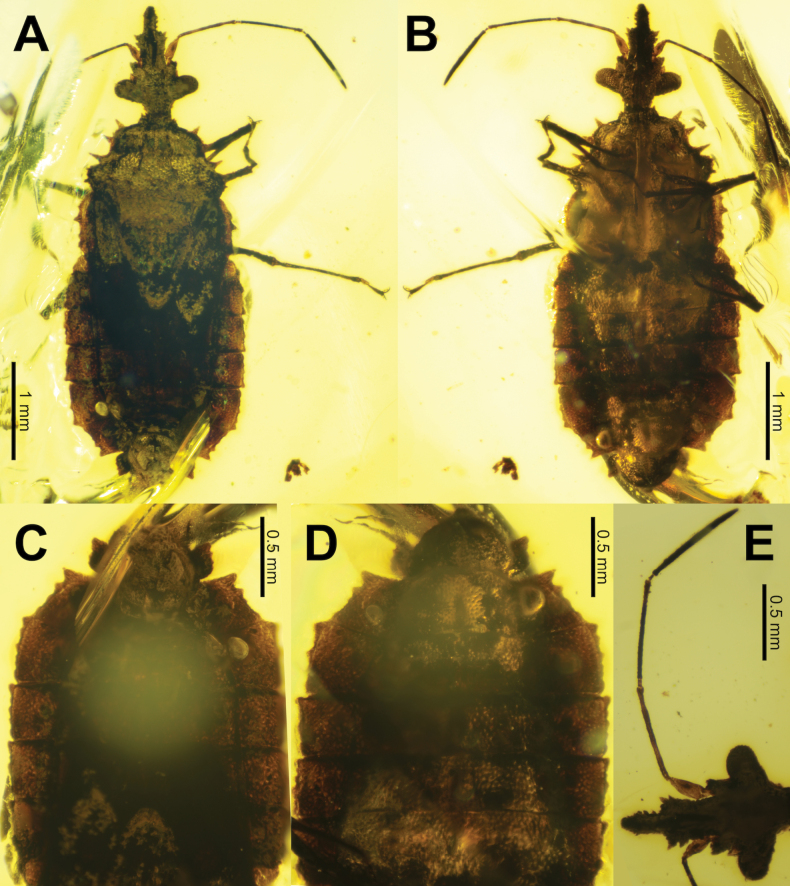
*Sauronaradusmeganae* gen. et sp. nov. holotype **A** dorsal habitus **B** ventral habitus **C** abdomen, dorsal **D** abdomen, ventral **E** right antenna, dorsal.

Distinguished from all known extant and extinct Aradidae by various features of the exceptionally long and thin antennae. Typically, aradid antennae are stockier and short, with antennomere lengths only 2×–10× the width, but in *Sauronaradus* gen. nov. antennomeres II, III, and IV have lengths ~20–22× their widths (Fig. 1E). Additionally, many aradids often have the terminal antennomere shorter than segments II or III, but in *Sauronaradus* gen. nov. the terminal antennomere is of a similar length and width to antennomeres II and III (Fig. 1E). *Sauronaradus* gen. nov. appears to possibly be related to the co-occurring Cretaceous species *Archemeziranuoxichenae* Heiss & Chen, 2023 and *Archemeziranuoyichenae* Heiss & Chen, 2023 as these also have a long and similarly shaped clypeus and long and slender antennae. *Sauronaradus* gen. nov. can be differentiated from *A.nuoxichenae* and *A.nuoyichenae* however by the terminal antennomere, which is longer than antennomeres II or III (versus *A.nuoxichenae* and *A.nuoyichenae* where the terminal antennomere is shorter than antennomeres II or III). Additionally, *Sauronaradus* gen. nov. can be differentiated from these species by the parallel-sided abdomen (versus broad and rounded) and the overall smaller size (~5 mm versus the massive 23.5 mm (*A.nuoyichenae*) and large 15.4 mm (*A.nuoxichenae*). *Sauronaradus* gen. nov. also shares characters with *Archearadus* Heiss & Grimaldi, 2001; such as the long clypeus, narrow neck, and spiniform tubercles of the pronotum.

#### Description.

Macropterous, medium body size ~5 mm (from the apex of the clypeus to the apex of the abdomen); body flat, lateral margins prominently marked with tubercles (with the pronotum margins with three large spiniform tubercles on each side ranging in length from 0.10–0.15 mm long; abdominal margins with granulation and minor tubercles); coloration dark brown.

***Head*.** Longer than wide, clypeus prominent and boxy, with two clearly defined widths, broad for the first half, then half as wide on the apex; antennae exceptionally long and thin with antennomere lengths ~20× the segment widths, segments II–IV of about equal length. Compound eyes large and bulging. Vertex of head with four spiniform tubercles transversing the compound eyes.

***Pronotum*.** A rounded isosceles trapezoid with three distinct widths from the anterior to the posterior. Lateral margin with three large spiniform tubercles. Surface marked throughout by small divots and four longitudinal carinae (the two in the center are more prominent and run the full length of the pronotum while the exterior carinae are less pronounced, only prominent on the posterior half).

***Scutellum*.** Rounded triangular, with the base width ca. equal to the length; surface flat without carinae, just slight granulation/divots.

***Legs*.** Armed with small granulation throughout (more prominent on femora, less so on the tibiae, with some of the more prominent nodes of the femora including a singular seta). Femora thicker than tibiae. Tibiae with dense, thick setae on the apical ends ventral surface. Tarsi two-segmented, claws with lemniscate pulvilli.

***Abdomen*.** Macropterous, but wing details are not discernable in the holotype. Only slightly wider than the thorax, with lateral margins that are subparallel; segments with weakly undulating margins creating three to five lumps with the posterior-most the most prominent and the others of a similar smaller size.

#### Remarks.

This new genus is tentatively placed in the subfamily ✝Archearadinae Heiss & Grimaldi, 2002. This subfamily lacks an apomorphic character that easily defines it, but instead has been defined by a set of characters from several extant subfamilies, which are held in a unique combination in the ✝Archearadinae. The features present in *Sauronaradus* gen. nov. which are known from the ✝Archearadinae are: clypeus long and prominent, open rostral atrium arising between the compound eyes not at the apex of the clypeus, abdominal tergites III and VI not fused but instead separated by a distinct suture, and the tarsi are two-segmented with claws bearing pulvilli. This tentative subfamilial placement is also supported by the morphological features shared between *Sauronaradus* gen. nov. and the genera *Archemezira* and *Archearadus*.

### ﻿ Sauronaradus meganae sp. nov.

Taxon classificationAnimaliaHemipteraAradidae

B0B5C9C1-779A-54B3-AD1E-F7E6FEA73551

https://zoobank.org/CC59C92B-2178-4C3C-B599-2114D420D433

Figs 1 , 2

#### Type material.

***Holotype***: specimen number IMQC-AMB-ara0001; Hukawng Valley, Myanmar, accession c. 2010; male; specimen deposited within the Montreal Insectarium, Montreal, Quebec, Canada (IMQC).

#### Taphonomy.

**Figure 2.**
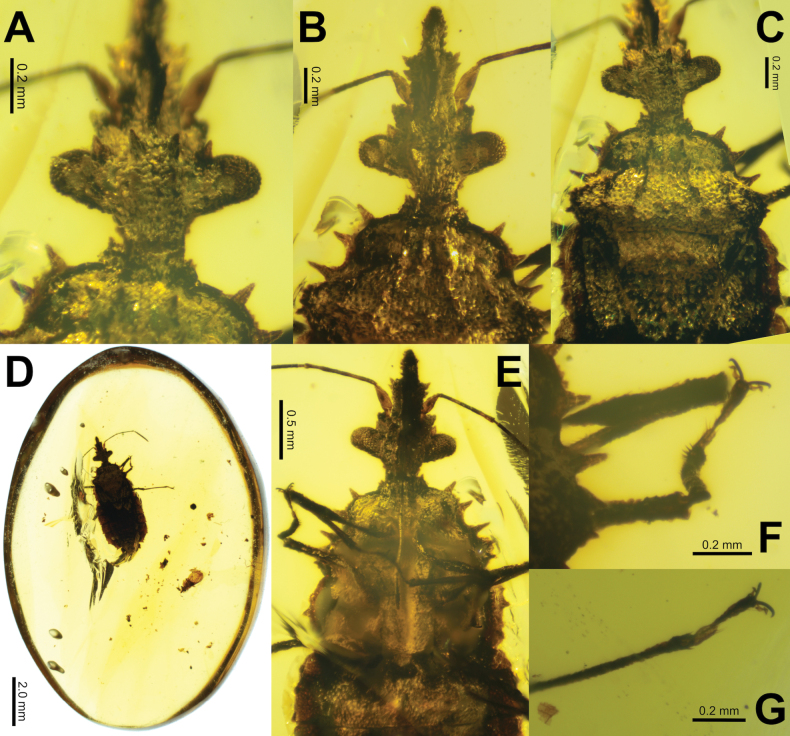
*Sauronaradusmeganae* gen. et sp. nov. holotype **A** anterior lit to highlight the spines of the head, dorsal **B** head through thorax, dorsal **C** pronotum, lit to highlight carinae, dorsal **D** full amber piece with holotype inclusion and *Cretopiesmasuukyiae* syninclusion to the lower right of *Sauronaradusmeganae* gen. et sp. nov. **E** head through thorax, ventral **F** pro- and meso- legs, mesotibia and tarsus visible, dorsal **G** right metatibia, dorsal.

Ovular piece of clear amber (~15.5 × 10.9 mm), with minimal debris obscuring the holotype (Fig. 2D). The holotype is wholly intact and appears to have little warping. Due to the dark coloration the wings are difficult to discern as they are tucked closely to the abdomen surface. One syninclusion is present, a slightly damaged *Cretopiesmasuukyiae* Grimaldi & Engle, 2008.

#### Type locality and horizon.

Latest Albian to lowermost Cenomanian (mid-Cretaceous), Hukawng Valley, northern Myanmar.

#### Etymology.

Patronym, named to honor Megan Solan, environmental toxicologist and entomologist. The first author wishes to thank Megan for her years of friendship and passion for entomology. Her enthusiasm for research is infectious and a positive driving force in the sciences.

#### Diagnosis.

Currently the genus is monotypic. See the above diagnosis of the genus for differentiation of this species from other aradids.

#### Description

(All measurements are in mm and based on the holotype). Macropterous male; large, 5.05 long, 1.88 wide; body flattened (Figs 1, 2).

***Head*** 1.16 long, 0.89 wide (across the compound eyes); dorsally and ventrally rough textured. Compound eyes strongly protruding (Fig. 2A). Ocelli absent. Clypeus long, protruding, and boxy, with two clearly defined widths, broad for the posterior half (loral lobe), then half as wide on the apical half (maxillary lobe; Fig. 2B). Dorsally there is a prominent anteclypeus on the posterior half (Fig. 2A). Clypeus loral lobe with two prominent apically pointing tubercles on each side; clypeus maxillary lobe with nodes on the dorsal and lateral surfaces (Fig. 2B). Ventral surface of head with nodes similar to the dorsal surface nodes, and a narrow median depression of gula (Fig. 2E). Bucculae not prominent. Open rostral atrium arising between the compound eyes, not at the apex of clypeus. Four, prominent, conical tubercles are present on the dorsal surface of the head. One pair are anteorbital tubercles present near the base of the compound eyes, and the second pair are situated evenly between the anteorbital tubercles (Fig. 2A). These four tubercles are of similar size and shapes. Head lacking prominent postocular lobes, instead immediately behind the compound eyes is the notably constricted postocciput, which is slightly narrower than the anterior of the pronotum.

***Antenna*** exceptionally long and thin with antennomeres II, III, and IV with lengths c. 20–22× their widths. Antenna with four antennomeres; basal antennomere (scape) the shortest, antennomere lengths: I: 0.27, II: 0.62, III: 0.70, IV: 0.74; antennomeres II, III, and IV tubiform; antennomeres II and III with surface granulation, and antennomere IV densely marked with setae throughout the surface with the seta length slightly less than the antennomere width, with the seta strongly angled apically (Fig. 1E). Antenniferous lobe prominent with a granular surface; stout, not projecting apically beyond the antennal insertion. Antennal insertion one-third of the way between the compound eyes and the apex of the clypeus (Fig. 2B). Rostrum with four segments extending to the posterior margin on the procoxal cavity (~1.73 mm long; Fig. 2E).

***Thorax*** pronotum (0.99 mm long, greatest width 1.44); roughly trapezoidal in shape (increasing in width caudally) but with somewhat undulating margins (approximate widths from the anterior to the posterior: 0.42 mm, 0.99 mm, 1.44 mm, 1.24 mm; Fig. 2C). Pronotum lateral margin armed on the anterior two-thirds with three prominent spiniform tubercles (anterior most 0.11 mm, middle 0.10 mm, posterior-most 0.15 mm long; Fig. 2B). The posterior third of the pronotum bulges out to a width of 1.44 mm, and this bulge is armed with several smaller tubercles (Fig. 2B). Pronotum surface punctate throughout, with two paramedial carinae on each side of the sagittal plane, the central pair of carinae extend the full length of the pronotum and diverge in a similar fashion to the pronotum width, while the exterior carinae are less pronounced and are more prominent on the posterior half (Fig. 2C). Carinae jagged in form, not uniform in intensity throughout the lengths, somewhat rising and falling in rough textures (Fig. 2C). Scutellum long and prominent, 1.24 mm wide, 1.16 mm long; dorsal surface with the anterior third slightly raised above the posterior two-thirds; surface flat but punctate throughout; apex broadly rounded. Prosternum; 0.49 mm long, surface punctate and marked with a moderate rostral groove which continues onto the mesosternum as a broader depression along the sagittal plane (Fig. 2E). Mesosternum maximum width 1.44 mm, length 0.51 mm; ventral surface punctate (Fig. 2E). Metasternum 0.51 mm long, 1.59 mm wide.

***Legs*** long and thin, all with slightly granular surfaces. Profemora 0.90 mm long, thinner on the proximal third. Leg lengths: protibiae 0.75 mm, mesofemora 0.92 mm, mesotibiae 0.60 mm, metafemora 1.16 mm, metatibiae 0.93 mm. Tibiae with sparse setae throughout, with longer and thicker setae on the distal ends (Fig. 2F). Tarsi with two tarsomeres; apical tarsomere is 1.5–2.0 times longer than the previous tarsomere; apical tarsomere thin proximally, widening notably for the proximal half, then a uniform broad width on the distal half (Fig. 2F, G). Apical tarsomere ventrally with few thin setae, previous tarsomere with thicker but sparse setae on the distal end (Fig. 2F). Tarsal claws long and simple; pulvilli approximately lemniscate, slightly shorter than tarsal claws (Fig. 2F, G).

***Wings*** fully developed, but details are indiscernible due to taphonomy of the holotype.

***Abdomen*** broad and flat, all surfaces punctate; length 2.41, greatest width 1.88 mm. Abdominal segment lengths: III = 0.57 mm, IV = 0.34 mm, V = 0.32 mm, VI = 0.37 mm, VII = 0.43 mm, VIII = 0.40 mm. Each abdominal segment has margins which gently undulate, with four or five humps, the posterior-most of which is on the posterior margin and larger than the others (Fig. 1C, D). Male terminalia broad and roughly bell shaped, surfaces rugose, a distinctly raised median ridge protrudes on the dorsal surface (Fig. 1C, D). Paratergite VIII rhomboid, angulated posteriorly, with the lateral margin slightly undulating (Fig. 1C, D).

## ﻿Discussion

Following this description of a new species, there are now 20 Aradidae species described from Cretaceous Kachin amber. Nine of the species have been described since 2020 suggesting that, even though this group is rarely found, it may have been diverse in the mid-Cretaceous, and we are only now beginning to understand this diversity. The morphological uniqueness of the species also demonstrates how little we know about the mid-Cretaceous flat-bug fauna. Goßner et al. (2007) demonstrated with extant fauna that aradid species require specific habitats to survive. As *Sauronaradusmeganae* gen. et sp. nov. and the two Cretaceous species of *Archemezira* all have long and thin antennae (when compared with the short, stout antennae typically seen in extant aradids), this suggests that perhaps these extinct species could have inhabited a different micro-habitat than modern aradids prefer. While modern aradids typically inhabit the bark of dead trees or leaf litter, with the flora of the Cretaceous significantly different than that of today this notable habitat difference could be correlated with these macro morphological differences in extant versus extinct aradid taxa. Hopefully, future research into Cretaceous aradid diversity will reveal overarching morphological trends.

## Supplementary Material

XML Treatment for Sauronaradus

XML Treatment for Sauronaradus meganae

## Footnotes

Tentatively assigned to this subfamily in the original description.

Originally described as Microaradus anticus Heiss & Poinar, 2012. Transferred to Cretopiesma in Azar et al. (2020).

Originally placed in Piesmatidae by Grimaldi and Engel (2008) and subsequently moved to general Aradidae by Cassis and Schuh (2010).

## ﻿Additional information

### Author ORCIDs

Royce T. Cumming https://orcid.org/0000-0001-7930-1292

Julia J. Mlynarek https://orcid.org/0000-0002-1569-9403

### Data availability

All of the data that support the findings of this study are available in the main text
